# Binase Immobilized on Halloysite Nanotubes Exerts Enhanced Cytotoxicity toward Human Colon Adenocarcinoma Cells

**DOI:** 10.3389/fphar.2017.00631

**Published:** 2017-09-12

**Authors:** Vera Khodzhaeva, Anna Makeeva, Vera Ulyanova, Pavel Zelenikhin, Vladimir Evtugyn, Martin Hardt, Elvira Rozhina, Yuri Lvov, Rawil Fakhrullin, Olga Ilinskaya

**Affiliations:** ^1^Institute of Fundamental Medicine and Biology, Kazan Federal University Kazan, Russia; ^2^Imaging Unit, Biomedical Research Center Seltersberg, Justus Liebig University Giessen Giessen, Germany; ^3^Institute for Micromanufacturing, Louisiana Tech University, Ruston LA, United States

**Keywords:** *Bacillus pumilus*, RNase, binase, halloysite, nanotubes, colon adenocarcinoma, cytotoxicity

## Abstract

Many ribonucleases (RNases) are considered as promising tools for antitumor therapy because of their selective cytotoxicity toward cancer cells. Binase, the RNase from *Bacillus pumilus*, triggers apoptotic response in cancer cells expressing *RAS* oncogene which is mutated in a large percentage of prevalent and deadly malignancies including colorectal cancer. The specific antitumor effect of binase toward RAS-transformed cells is due to its direct binding of RAS protein and inhibition of downstream signaling. However, the delivery of proteins to the intestine is complicated by their degradation in the digestive tract and subsequent loss of therapeutic activity. Therefore, the search of new systems for effective delivery of therapeutic proteins is an actual task. This study is aimed to the investigation of antitumor effect of binase immobilized on natural halloysite nanotubes (HNTs). Here, we have developed the method of binase immobilization on HNTs and optimized the conditions for the enzyme loading and release (i); we have found the non-toxic concentration of pure HNTs which allows to distinguish HNTs- and binase-induced cytotoxic effects (ii); using dark-field and fluorescent microscopy we have proved the absorption of binase-loaded HNTs on the cell surface (iii) and demonstrated that binase-halloysite nanoformulations possessed twice enhanced cytotoxicity toward tumor colon cells as compared to the cytotoxicity of binase itself (iv). The enhanced antitumor activity of biocompatible binase-HNTs complex confirms the advisability of its future development for clinical practice.

## Introduction

Ribonucleases (RNases) have the potential to destroy RNA in cancer cells and generate signals leading to the cancer cell death. The downstream mechanisms of RNase cytotoxicity may induce regulatory activity of RNA hydrolysis products, as well as selective suppression of certain oncogenes. Representatives of eukaryotic and prokaryotic RNases display antitumor properties. Among them are onconase, a cytotoxic RNase derived from leopard frog oocytes or early embryos, bovine seminal RNase, eosinophil cationic protein, *Bacillus pumilus* RNase binase, and *B. amyloliquefaciens* RNase barnase (Makarov et al., 2008; Ulyanova et al., 2011; Mitkevich et al., 2014; Olombrada et al., 2017; Vert et al., 2017). Since the RNase inhibitor presented in human tissues blocks the catalytic activity of mammalian RNases, phylogenetically distant enzymes have an advantage for creating new antitumor drugs. Onconase has been applied to the treatment of malignant lung mesothelioma and metastatic kidney cancer in clinics. Unfortunately, the clinical trials did not lead to the desired increase in life expectancy (Vogelzang et al., 2001). Currently, onconase is tested as a part of combination therapy against lung cancer (Shen et al., 2016).

Over the last years, research interest in binase is constantly being increased because of selective cytotoxicity of this enzyme toward certain cancer cells. Binase inhibits proliferation of human leukemic Kasumi-1 cells, lung adenocarcinoma cells, ovarian and breast cancer cells (Mitkevich et al., 2011; Cabrera-Fuentes et al., 2013; Garipov et al., 2014; Zelenikchin et al., 2016). The sensitivity of cancer cells to binase toxic action depends on the expression of *ras, kit*, AML1-ETO and FLT3 oncogenes, but does not depend on the expression of *fms* and *src* oncogenes (Ilinskaya et al., 2001, 2016; Mitkevich et al., 2011, 2013b). The ability of binase to reduce primary tumor and metastasis growth in animals with induced tumors was demonstrated on Lewis lung carcinoma, lymphosarcoma RLS40 and melanoma B-16 (Mironova et al., 2013). An important fact is the absence of necrosis induced by binase in cell cultures as well as *in vivo* for the entire organism in murine tumor model. Additionally, binase triggers regenerative processes in the liver of mice with Lewis lung carcinoma and has no general toxic effect on the organism in normal mice (Mironova et al., 2013). The possible mechanism of binase cytotoxic action includes enzymatic degradation of RNA molecules with generation of a novel set of small regulatory RNAs (miRNAs) and startup of apoptotic pathways (Mitkevich et al., 2013a). Moreover, the direct binding of binase to the RAS protein leading to inhibition of RAS/MAPK/ERK proliferative signaling was found (Ilinskaya et al., 2016).

Mutations in RAS occur in a large percentage of prevalent and deadly malignancies including melanoma, lung adenocarcinoma, colorectal cancer (CRC), pancreatic cancer, acute leukemias, and others. Targeting RAS remains a critical priority for cancer therapy but an effective, approved treatment option has remained elusive in any cancer to date (Johnson and Puzanov, 2015). According to the International Agency for Research on Cancer reports, over 500,000 of new CRC cases are registered each year in the world. RAS point mutations in codons 12 and 13 of exon 2 leading to activation of RAS/MAPK/ERK signaling pathway, are most common in CRC and indicates a poor prognosis of the disease. Activation of KRAS due to mutations eliminates the effect of inhibition of EGFR by monoclonal antibodies. The specific antitumor effect of binase toward RAS-mutant malignancies in general and CRC in particular supports the development of anticancer therapies based on this RNase.

Delivery of proteins to the intestine is complicated by their degradation in the digestive tract and subsequent loss of therapeutic activity. The search of the new effective systems for drug delivery takes the leading place in pharmaceutics. The loading of various therapeutics, e.g., cytotoxic anticancer drugs, into neutral small carrier may help to avoid their destruction and possible side effects. Moreover, these nanoscale vehicles increase the concentration of an active therapeutic agent in targeted tumor cells or tissues through sustained release. Natural halloysite nanotubes (HNTs) appear to be promising agents for targeted drug delivery. HNTs were used for effective loading of nanotubes with various therapeutic agents, antiseptics, proteins, as well as with protective chemical inhibitors (Joussein et al., 2005; Du et al., 2010; Liu et al., 2013; Lvov et al., 2014, 2016). Recently, it was demonstrated that loading of enzymes into the halloysite inner lumen enhances their stability and provides for longer biocatalytic functionality (Sun et al., 2017). The protein-drug immobilization on nanotube carrier may also promote intercellular drug delivery (Dzamukova et al., 2015; Yendluri et al., 2017).

Here, we focused on the study of potential antitumor action of binase immobilized on HNTs. First, we have developed the method of binase immobilization on nanotubes and optimized the conditions for the enzyme loading/release. Taken into account the specific cytotoxic activity of binase toward RAS-expressing malignances, we have chosen human colon cancer cells as an object for the experimental investigation of binase antitumor effects. Second, we found the concentration of pure HNTs which is non-toxic for cells and allows to distinguish HNTs-induced and binase-induced cytotoxic effects. Using dark-field and fluorescent microscopy we revealed the absorption of binase-HNTs complex on the cell surface. Finally, we have demonstrated that binase-HNTs complex possessed enhanced cytotoxicity toward tumor colon cells compared to cytotoxicity of the enzyme itself. Our results provide a basis for perspective development of binase-halloysite nanoformulations for therapy of CRC.

## Materials and Methods

### Binase

The guanyl-preferring RNase from *B. pumilus*, binase (12.2 kDa, 109 amino acid residues, pI 9.5), was isolated from the culture fluid of native binase producer as homogenous protein using the three-step procedure described earlier (Dudkina et al., 2016). The binase catalytic activity (1.4 × 10^6^ units/mg) was determined by measurement of high-polymeric yeast RNA hydrolysis products according to modified method of Kolpakov and Il’inskaia (1999).

### Nanotubes

High purity (98%) HNTs were provided by Applied Minerals Inc. (United States). HNTs are natural aluminosilicate clay with 50–60 nm external diameter, 12–15 nm inner lumen diameter and of 500–800 nm length (Shutava et al., 2014). The outer surface of HNTs is composed of silicon oxide (SiO_2_) and has a negative charge at pH above 4 with typical electrical zeta-potential of -30 mV; the inner tube surface of aluminum oxide (Al_2_O_3_) is positively charged with potential of +21 mV at pH 4–8.5 (Kamble et al., 2012; Shutava et al., 2014). HNTs have a high surface area of 60–70 m^2^/g and are easily dispersed forming colloids in aqueous solutions, PBS or serum as physiological pH (Wei et al., 2014).

### Immobilization of Binase on Nanotubes

Halloysite nanotubes were washed with a sterile filtered 70% ethanol and thoroughly vortexed (V-1 plus, Biosan, Latvia) until a homogeneous suspension, and then sonicated in ice for 5 min, 35 kHz, 130 W (Sapphire, Russia) to disintegrate the tubes aggregates. Afterward the tubes were centrifuged (Eppendorf 5415R, Eppendorf, Germany) for 5 min at 4300 *g* and washed with sterile water to remove ethanol. The following solutions were used to load the enzyme on HNTs: (1) water, pH 5.5 (Milli-Q Direct 8, Millipore, France); (2) 0.25 M Tris-HCl buffer, pH 8.5; (3) 10 mM Na-acetate buffer, pH 5.0; (4) 10 mM Na-phosphate buffer, pH 7.0. The protein concentration was measured using absorption spectrophotometry at 280 nm (SmartSpec Plus, Bio-Rad, United States).

**FIGURE 1:**
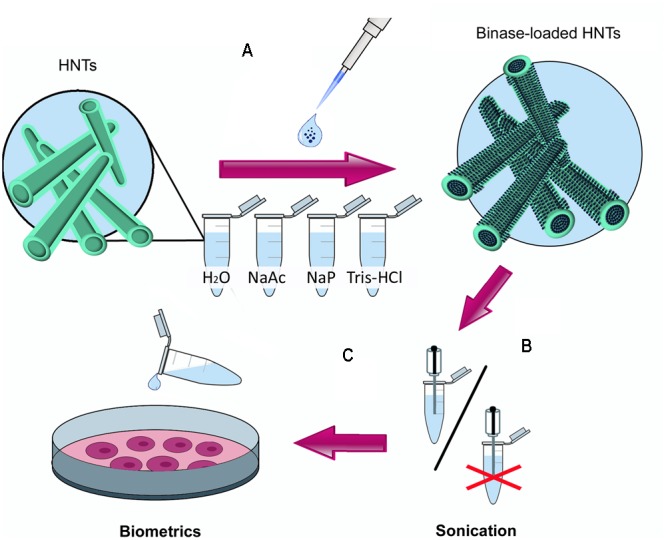
The scheme of the experimental work. **(A)** selection of conditions for loading of antitumor binase on halloysite nanotubes (HNTs) using solutions with different pH (NaAc, 10 mM Na-acetate buffer, pH 5.0; H_2_O, water of Milli-Q grade, pH 5.5; NaP, 10 mM Na-phosphate buffer, pH, 7.0; Tris-HCl, 0.25M Tris-HCl buffer pH 8.5); **(B)** optimal binase release from HNTs at pH 7.0. Due to the absence of difference in the quantity of released enzyme between sonicated and unsonicated samples the sonication step was excluded; **(C)** treatment of human colon adenocarcinoma cells Colo 320 with binase-loaded HNTs.

Prepared suspension of HNTs (10 mg/ml) was mixed with binase (1 mg/ml), vortexed (V-1 plus, Biosan, Latvia) and incubated for 2 h with gentle shaking (Mini Rocker-Shaker MR-1, Biosan, Latvia) at 4°C. The zeta-potential of binase-loaded HNTs was monitored using a Zetasizer Nano ZS instrument (Malvern, United Kingdom). To estimate the quantity of immobilized binase, protein concentration (*A*_280_) and RNase catalytic activity of the supernatant obtained after centrifugation (5 min, 4300 *g*, Eppendorf 5415R, Germany) were measured. The same procedures were carried out for bovine serum albumin (Amresco, United States) as control protein without cytotoxic activity. Enzyme release after 24 h incubation was carried out using buffers of different ionic strength and pH. To reduce the enzyme release, binase-loaded HNTs were coated with dextrin (Sigma–Aldrich, United States) according to the method described earlier (Dzamukova et al., 2015). **Figure 1** shows all steps used for binase loading onto nanotubes and subsequent enzyme release in cultural medium of colon adenocarcinoma cells.

### Cell Culture

Colon adenocarcinoma cells (Colo 320) were obtained from Russian cell culture collection (Saint-Petersburg, Russia). Cells were grown in RPMI 1640 medium supplemented with penicillin (100 U/mL), streptomycin (100 U/mL), 2 mM glutamine (Sigma-Aldrich, United States) and 10% fetal bovine serum (HyClone, United States) at 37°C in a humidified atmosphere with 5% CO_2_. Cells were seeded into 96-well or 24-well plates, tested samples (binase 100 μg/ml, albumin 100 μg/ml, HNTs 625 μg/ml, HNTs loaded with binase 100 μg/ml and HNTs loaded with albumin 100 μg/ml) were dissolved in fresh medium and added into plates in accordance with assay procedure. Triton X-100 (1% solution) was used as a positive control. Experiments were performed in triplicates with five parallel measurements.

### MTT-Assay

Cell viability was measured according to mitochondrial dehydrogenase activity tested by standard procedure based on the reduction of MTT tetrazolium dye. Cells (10^4^ per well in 96-well plate, CELLTREAT Scientific Products, United States) have grown overnight, then cultural fluid was discarded and fresh medium with test samples was added. After 24 h, formazan absorption was measured (xMark, Bio-Rad, United States).

### Fluorescence Microscopy

Colo320 cells were seeded into 24-well plates (7 × 10^4^ per well) and incubated for 24 h. Then, HNTs (625 μg/ml), free and immobilized on HNTs binase (100 μg/ml) were added followed by staining with 3,3′-dihexyloxacarbocyanine iodide (DiOC_6_) and propidium iodide (PI). Measurement of the fluorescence intensity was performed on an Axio Observer A1 microscope (ZEISS, Germany), λEm = 520 nm for DiOC_6_ and λEm = 670 nm for PI. The percentage of living cells was counted using ImageJ Cell Counter Software (NIH, United States).

### Dark-Field Microscopy

The distribution of HNTs in the cells was analyzed using enhanced dark-field microscopy. Colo320 cells were grown on glass cover slips, HNTs were added after 24 h, then cells were stained with 4,6-diamidino-2-phenylindole (DAPI) for 20 min. Afterward samples were fixed by ice-cold acetone (-20°C). Cover slips with fixed samples were attached to the microscope slides by the mounting media. The CytoViva enhanced dark-field condenser (CytoViva, United States) fitted to an Olympus BX51 upright microscope (Olympus, Japan) was used to visualize the HNTs in human cells with DAPI-stained nuclei.

### Scanning Electron Microscopy

All specimens were mounted on SEM-holders left in ambient air for 5 days, lightly gold-sputtered (Balzers SCD 004, Oerlikon Balzers, Liechtenstein) and inspected in a FEG scanning electron microscope DSM982 (ZEISS, Germany) at 10 kV. Images were recorded using a secondary electron (SE)-detector with the voltage of the collector grid biased to +300 V in order to improve the signal-to-noise ratio and to reveal optimal topographical contrast. The settings for SEM, including tilt angle, spot size, scanning mode were kept constant for all sample groups.

### Transmission Electron Microscopy

A droplet of diluted HNTs or HNTs loaded binase suspension was placed onto carbon-coated grids and left to evaporate. Specimens were inspected using a Hitachi HT7700 Exalens transmission electron microscope (Hitachi High-Tech Science Corporation, Japan) at resolution 1,4 Å. TEM bright field images were recorded at 100 kV accelerating voltage using a AMT XR-81 CCD camera (3296 × 2742, 8 megapixel, 5,5 μm pixel size).

### Statistics

Statistical data analysis and plotting were performed by means of GraphPad Prism7 software (United States). The statistically significant level was taken as *p* ≤ 0.05.

## Results

**FIGURE 2:**
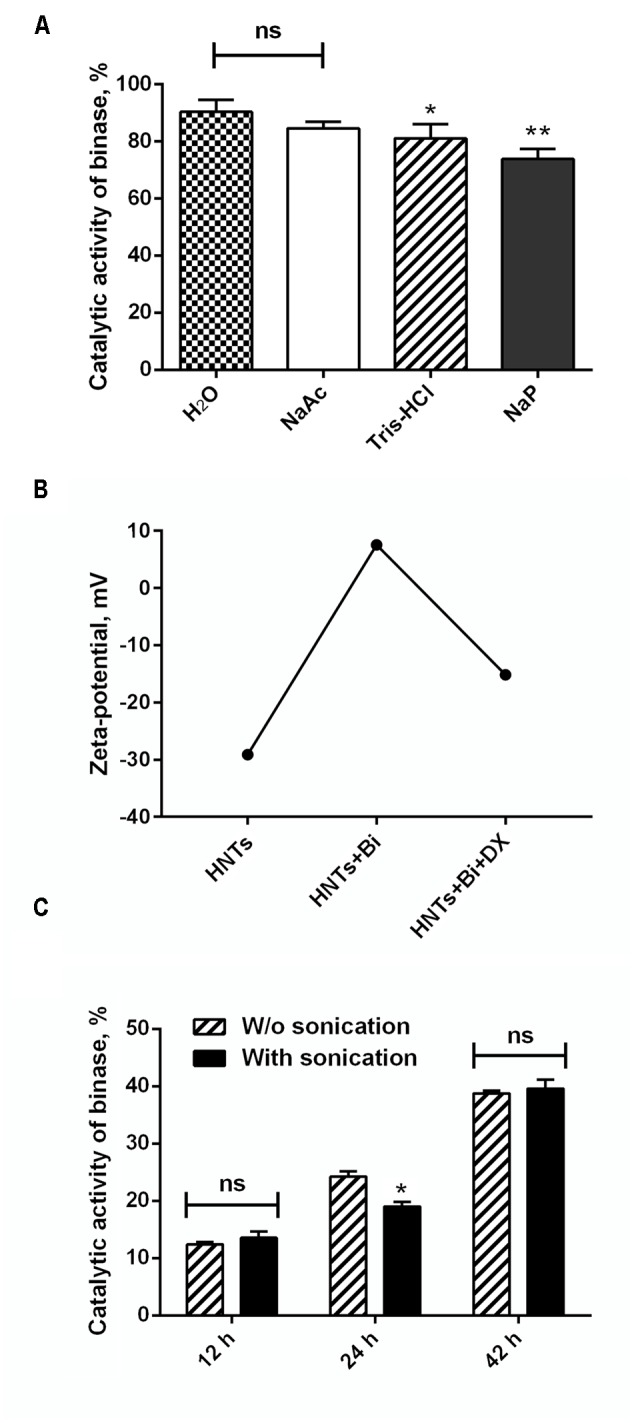
Characterization of binase loading on and release from HNTs. **(A)** The quantity of binase loaded onto HNTs at various pH (NaAc, 10 mM Na-acetate buffer, pH 5.0; H_2_O, water of Milli-Q grade, pH 5.5; NaP, 10 mM Na-phosphate buffer, pH, 7.0; Tris-HCl, 0.25M Tris-HCl buffer pH 8.5). **(B)** Zeta-potential of HNTs, binase-loaded HNTs and binase-loaded HNTs coated with dextrin. **(C)** Time-dependent release of binase from HNTs at neutral pH with and without sonication. Initial enzyme concentration in solutions was 1 mg/ml. The catalytic activity of binase in each solution is taken for 100%. Data represent mean ± SEM of three independent experiments; ^∗^*P* < 0.05, ^∗∗^*P* < 0.01 vs. control, ns, non-significant.

To find the optimal method for the immobilization of binase on nanotubes, we tested different solutions for enzyme loading (**Figure 1A**). During the selection of RNase loading conditions we found that 90% of the enzyme was adsorbed at pH 5.5, which corresponds to the pH of distilled water prepared by a Milli-Q water purification system (Millipore). Loading of binase on HNTs in Na-acetate buffer allowed us to immobilize 85% of binase from the solvent. Enzyme loading in the neutral (Na-P phosphate buffer, pH 7.0) and weakly alkaline solutions (Tris-HCl buffer, pH 8.5) proved to be less effective: only 75% of protein was loaded in the Na-P phosphate buffer and 80% in the Tris-HCl buffer (**Figure 2A**). It could be explained by the partial neutralization of binase cationicity (pI 9.5) at alkaline conditions. Therefore for further experiments we used a Milli-Q water pH 5.5 as a loading solution. Nevertheless, binase remained positively charged molecule that allowed adsorption at the outermost of the negative HNTs. The total loading of the halloysite estimated by dividing total mass of the binase-adsorbed HNTs onto halloysite mas was approximately 7 wt %.

**FIGURE 3:**
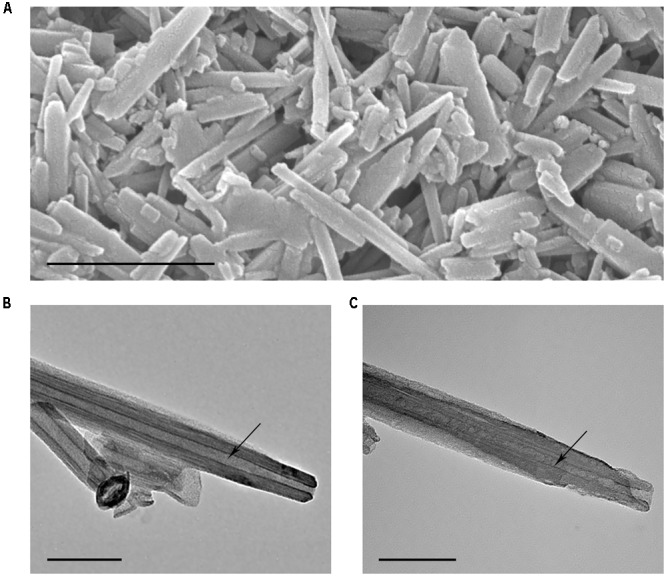
Electron microscopy images of HNTs. SEM image of pure HNTs **(A)**, TEM image of pristine halloysite **(B)** and binase-loaded HNTs **(C)**. Arrows indicate the lumen of pristine nanotubes **(B)** and nanotubes loaded with binase **(C)**. Scale bar in **(A)** is 1 μm, in **(B,C)** is 100 nm.

Penetration of binase into nanotube lumen was demonstrated by TEM microscopy. SEM image of HNTs characterized the purity of nanotube preparation used in this study (**Figure 3A**). Comparison of pure and binase-loaded HNT images allowed us to conclude that enzyme is located inside of the nanotubes’ lumen as small irregular structures (**Figures 3B,C**). To check a possibility of binase to be located on the surface of nanotubes, we measured zeta-potential of HNTs before and after binase loading (**Figure 2B**). We have found that zeta-potential of binase-loaded nanotubes is slightly positive (+6 mV) if compared with negatively charged non-loaded nanotubes (-29 mV). Because of limited enzyme release during the measurement, the increase of zeta-potential reflects also the interaction of cationic enzyme with nanotube surface. Binase-loaded nanotubes were coated with dextrin via vacuum-facilitated deposition resulting in formation of physically adsorbed dextrin layer on HNTs. If binase-loaded nanotubes were coated by neutral dextrin, the partial return of zeta-potential to the previous negative value due to the reduction of enzyme release by dextrin stoppers should be observed. Indeed, in the variant with dextrin-coated binase-loaded HNTs zeta-potential was decreased (-15 mV) as compared to the variant without dextrin (+7 mV) indicating that dextrin doesn’t allow the enzyme to be released from HNTs lumen (**Figure 2B**).

Binase release from HNTs occurred gradually starting from 12 h of incubation. The enzyme yield at pH 7 after 42 h of incubation measured according to the catalytic activity and protein content reached 40% from the loaded enzyme (**Table 1**). The release of binase at acidic and alkaline pH at that time point was 25 and 21.8%, correspondingly. Thus, an optimal environment for the enzyme loading was slightly acidic while an optimal environment for its release was the neutral one (**Table 1**). To enhance the enzyme release from clay nanotubes, ultrasonic sound was applied. We used both options, with and without sonication, and found that sonication did not result in a statistically significant increase in the enzyme yield after 12 and 42 h of incubation and only a minor difference (about 6%) was observed after 24 h of incubation (**Figure 2C**).

**Table 1:** Release of binase from nanotubes vs. time and solution pH measured by protein concentration and its catalytic activity, %.

	Time, h					
	12		24		42	
pH	Protein concentration	Catalytic activity	Protein concentration	Catalytic activity	Protein concentration	Catalytic activity
5.0	0	0	10	10.5	23.7	25
5.5	0	0	17	14.5	25	22.7
7.0	15	12.375	26	24.25	35.87	40
8.5	8	7.6	15	14.9	20	21.8

*The initial protein absorption value and catalytic activity of binase at concentration 1 mg/ml were taken for 100%. The values are presented as Rms of three independent experiments with standard deviation σ ≤ 10%.*

**FIGURE 4:**
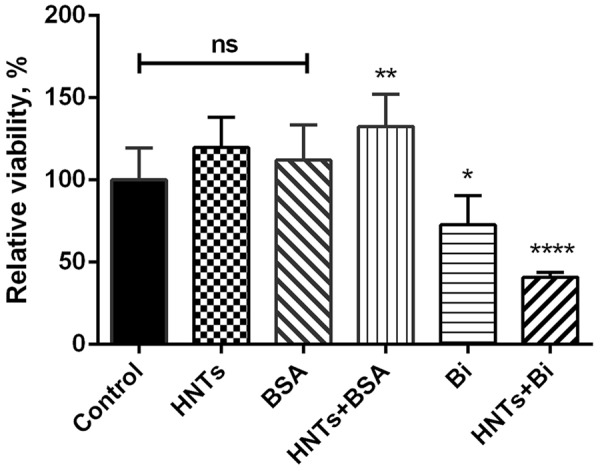
Cytotoxicity of binase-loaded HNTs toward human colon adenocarcinoma Colo320 cells. Viability of cells in the absence of studied agents was taken as 100%. Data represent mean ± SEM of three independent experiments; ^∗^*P* < 0.05, ^∗∗^*P* < 0.01, ^∗∗∗∗^*P* < 0.0001 vs. control, ns, non-significant.

The cytotoxicity of HNTs was studied on human colon adenocarcinoma cell line Colo 320. Binase-free HNTs at concentration up to 625 μg/ml did not exert any cytotoxicity during 24 h of treatment, the viability of cells was the same like of non-treated cells (**Figure 4**). As a control we used HNTs loaded with bovine serum albumin (BSA, 100 μg/ml). The optimal quantity of loaded BSA was 90%. Its release at neutral pH was about 35%. BSA-loaded HNTs as well as free BSA did not exhibit any cytotoxic effects. Addition of HNTs loaded with 100 μg/ml binase to the cell culture decreased percentage of living cells in population by 60%. Binase alone at the same concentration reduced cell viability by 25%. Therefore, binase immobilization on the halloysite significantly increased the enzyme cytotoxicity toward Colo 320 cells (**Figure 4**).

**FIGURE 5:**
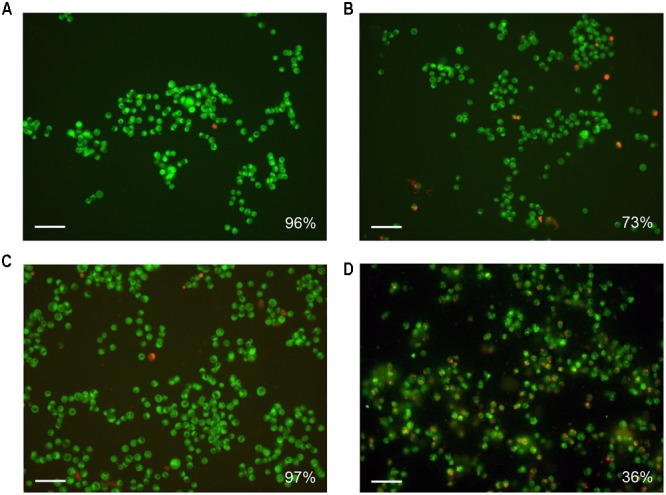
Fluorescence microscopy of Colo 320 cells after 24 h of incubation **(A)** and simultaneous treatment by binase **(B)**, HNTs **(C)**, and HNTs loaded with binase **(D)**. Cells were stained with 3,3′-dihexyloxacarbocyanine iodide (DiOC_6_) and propidium iodide, viable cells show green fluorescence, dead cells have red emission color. The number of viable cells is expressed as a percentage of the total cell number. 100 000 cells in each variant were taken for 100%. Scale bar is 50 μm.

**FIGURE 6:**
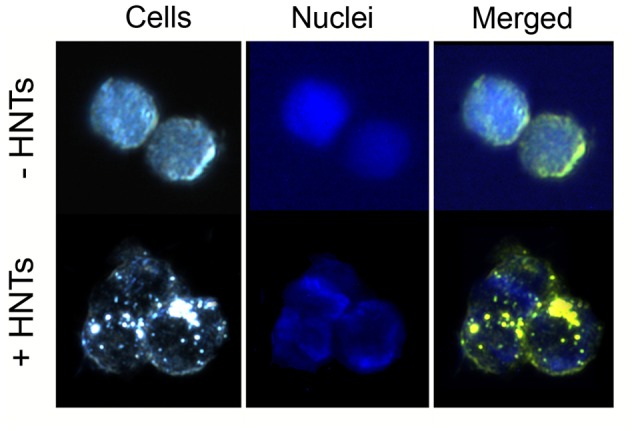
Microscopic visualization of Colo 320 cells after 24 h of incubation with 100 μg/ml HNTs. Dark-field image of cells **(left)**, fluorescent image **(middle)**, merge image **(right)**. Cells nuclei are stained with DAPI.

The results of cytotoxicity assay were confirmed by the data obtained by fluorescence microscopy: the amount of living cells without treatment and treated for 24 h by pristine HNTs was 96 and 97%, correspondingly (**Figures 5A,C**). Binase and binase-loaded HNTs reduced the number of viable cells by 27 and 64%, respectively (**Figures 5B,D**). Using dark field and fluorescent microscopy we detected bright spots surrounding the cells which demonstrated location of HNTs on the cell surface (**Figure 6**).

## Discussion

Currently, a number of gene therapy strategies are being evaluated in patients with cancer. These include manipulating cells to gain or lose function. Inactivation of genes which cause uncontrolled growth of cancer cells was carried out by many approaches including gene silencing by anti-sense oligonucleotides, short interfering RNA and short hairpin RNA that target mRNA of these genes. Disruption of proliferative pathways can be also performed by inactivation of signaling proteins. It is especially important to block the initial stages for prevention of signaling expansion. RAS proteins are anchored on the cytoplasmic side of the cell membrane and play a central role in cell signaling transferring signals from the cell membrane to the nucleus. Oncogenic mutations in the *ras* gene are present in approximately 30% of all human cancers. We propose that binase antitumor action could contribute to therapy of CRC and other malignancies expressing mutant KRAS due to RNA targeting followed by formation of small interfering RNAs (Mitkevich et al., 2010) and inhibition of RAS/MAPK/ERK proliferative signaling by direct binding of binase to KRAS oncogene (Ilinskaya et al., 2016).

Peroral administration of therapeutic proteins meets a number of difficulties associated with the inactivation by proteases and the loss of enzyme activity. Not only proteins, but also other therapeutic agents need prolonged effectiveness in organism. Therefore, the application of liposomes, micelles (Joo et al., 2013), supramolecular containers (Cao et al., 2013) and magnetic nanoparticles (Fan et al., 2013; Lee et al., 2013) as carriers for drug delivery deserves a special interest. For example, polyelectrolyte micro/nano capsules were successfully exploited for targeted and controlled anticancer drug delivery (Ariga et al., 2011; Deng et al., 2013). There were multiple efforts to use carbon nanotubes for drug delivery; however, nanotubes were found to be cancerogenic, which provide concerns over their usage in medicine (Alshehri et al., 2016). Different types of liposomes are already widely used in clinical practice. A chemotherapeutic agent doxorubicin (Caelyx^®^ in Europe and Doxil^®^ in the United States) is extensively used for the treatment of malignant neoplasms. Loading of doxorubicin into liposomes resulted in increased antitumor effect of the drug (Davies et al., 2004). Alternative delivery system based on chitosan nanoparticles provided loading of up to 70% of doxorubicin (Soares et al., 2016), whereas delivery system based on modified single-walled carbon nanotubes allowed not only increasing of the doxorubicin efficiency during prolonged drug release, but also reduced the degree of its general toxicity (Farvadi et al., 2016).

A number of studies demonstrated biocompatibility of HNT which was assessed for microorganisms, nematodes and human cells (Vergaro et al., 2010; Zhang et al., 2014; Fakhrullina et al., 2015). According to Vergaro et al. (2010) and Lai et al. (2013), HNTs were non-toxic to the cervix carcinoma HeLa and breast adenocarcinoma MCF-7 cell lines at concentrations of 100 μg/ml. It was shown that curcumin-loaded HNTs induce a high cytotoxicity toward two different cell lines of hepatocellular carcinoma compared to the free curcumin or pristine HNTs (Massaro et al., 2016a). A dual drug-loaded delivery system based on HNTs and carbohydrate functionalized cyclodextrin demonstrated an improved cytotoxicity against 8505c thyroid carcinoma cell lines compared to free drugs (Massaro et al., 2016b).

Based on the accumulated data on the high therapeutic potential of RNases, we chose binase, secreted guanyl-preferring RNases of the N1/T1/U2 family (EC 3.1.27.3) (Ulyanova et al., 2011, 2016), for loading to the clay nanotubes. We have selected the conditions in which the enzyme was almost completely loaded to HNTs (**Figure 2A**). Binase isoelectric point is 9.5 and enzyme is cationic at pH 6–8, therefore, the positive charge on the halloysite tube interior unlikely will facilitate enzyme loading into the lumen. However, the size of binase molecule (about 3.3 nm in diameter) calculated with the help of CalcTool (CalcTool: Protein size calculator^1^) allows its penetration into HNT lumen (12–15 nm inner lumen diameter). Due to the patchy nature of the protein globule charge, the location of binase inside of HNT is also possible. Probably, hydrophobicity can play a much greater role in protein going inside the HNTs. Binase has a hydrophobic segment, which is sterically available for the hydrophobic interaction with a tumor cell membrane (Shirshikov et al., 2013). As it was shown by TEM technic, a part of binase is actually located inside of HNT (**Figure 3C**). However, its adsorption on the tube’s negative surface cannot be excluded. The increase of negative zeta-potential of HNTs up to positive values for HNTs loaded with binase supports the superficial location of enzyme (**Figure 2B**). On the other hand, this result can also be explained by the partial release of cationic binase from HNT lumen. Indeed, dextrin stoppers had not allowed the enzyme to be released from HNTs lumen (**Figure 2B**). We assume that binase immobilization inside halloysite lumen will prevent its decomposition by cellular proteases and provide a prolonged action of this enzyme, thus reducing the drug dosing frequency. Here, using MTT-assay and double fluorescent staining, we have showed that viability of Colo320 cell line was maintained at 100% level after addition of pristine HNTs at as high concentration as 625 μg/ml (**Figures 4, 5C**). For hepatoma HepG2 cells and HCT116 colon carcinoma cells HNTs were found to exert significant anti-proliferative activity only at 1000 μg/ml concentration (Ahmed et al., 2015). Therefore, the HNTs themselves in the concentrations up to 1000 μg/ml may be applied as nanovehicle for delivery of antitumor protein agents. It should be noted that considerable success was achieved at peroral application of modified HNTs for protection of livestock against the harmful effects of mycotoxin zearalenone presented in grain feed (Zhang et al., 2014).

In our study binase immobilized on HNTs retained its catalytic activity measured by enzyme release at optimal conditions by pH 7 for 1 month. It corresponds to the published data that various enzymes such as invertase, cellulase, peroxidase, and laccase retained their catalytic activity for a long time after loading into nanotubes (Lundqvist et al., 2004; Safari et al., 2005; Tully et al., 2016).

We demonstrated that binase-halloysite complex doubled anticancer efficiency of binase due to its perfect absorption by cells (**Figure 6**) and longer release. In anticancer nanoformulation, we characterized HNT loaded with binase as a highly cytotoxic complex which reduced the viability of human colon adenocarcinoma cells by 60%, whereas binase alone was found to be twice less effective (**Figure 4**). The enhancement of binase antitumor activity by complexation with HNTs is an essential step for the development of tumor treatment strategies. A loading of cytotoxic binase into clay nanotubes may be considered as a promising tool for colorectal cancer treatment in the form of rectal suppositories. Taken into account that halloysite is not biodegradable, external applications of binase-loaded HNTs will be the most secure mode and could be tested for treatment of malignant skin rebirth.

The unique abilities of microorganisms to synthesize practically useful substances (antibiotics, cytostatics, proteins, etc.) serve as an inexhaustible reserve for the development of new therapeutics of natural origin. The priority results obtained by us on the cytotoxic effect of binase-HNTs complex toward colon carcinoma cells emphasize once again the promising potential of microbial RNases in the development of antitumor drugs.

## Author Contributions

VK, VU, PZ, RF, YL, and OI planned experiments. VK, AM, VE, MH, and ER performed experiments. VK, AM, VU, PZ, MH, YL, RF, and OI analyzed data. PZ, MH, and RF contributed reagents. VK, VU, YL, RF, and OI wrote the paper.

## Conflict of Interest Statement

The reviewer HR declared a shared affiliation, with no collaboration, with one of the authors, MH, to the handling Editor. The other authors declare that the research was conducted in the absence of any commercial or financial relationships that could be construed as a potential conflict of interest.

## Footnotes

1 http://www.calctool.org/CALC/prof/bio/protein_size
